# The Role of Memory Traces Quality in Directed Forgetting: A Comparison of Young and Older Participants

**DOI:** 10.5334/pb.au

**Published:** 2014-06-23

**Authors:** Fabienne Collette, Julien Grandjean, Caroline Lorant, Christine Bastin

**Affiliations:** 1 Cyclotron Research Centre, University of Liège, Belgium; 2 Department of Psychology: Cognition and Behavior, University of Liège, Belgium

**Keywords:** aging, inhibition, memory, directed forgetting

## Abstract

A reduced directed-forgetting (DF) effect in normal aging has frequently been observed with the item method. These results were interpreted as age-related difficulties in inhibiting the processing of irrelevant information. However, since the performance of older adults is usually lower on items to remember, the age effect on DF abilities could also be interpreted as reflecting memory problems. Consequently, the present study aimed at investigating the influence of memory traces quality on the magnitude of the DF effects in normal aging. We predicted that increasing the quality of memory traces (by increasing presentation times at encoding) would be associated with attenuated DF effects in older participants due to the increased difficulty of inhibiting highly activated memory traces. A classical item-method DF paradigm was administered to 48 young and 48 older participants under short and long encoding conditions. Memory performance for information to memorize and to suppress was assessed with recall and recognition procedures, as well as with a Remember/Know/Guess (RKG) paradigm. The results indicated that, when memory traces are equated between groups, DF effects observed with the recall, recognition and RKG procedures are of similar amplitude in both groups (all ps>0.05). This suggests that the decreased DF effect previously observed in older adults might not actually depend on their inhibitory abilities but may rather reflect quantitative and qualitative differences in episodic memory functioning.

## Introduction

Forgetting of information, often conceptualized and perceived as a memory failure, can nevertheless, in some circumstances, be adaptive and lead to a better memory functioning. More specifically, forgetting permits us to update our memory content, by processing current information without interference from no longer relevant information or by inhibiting closely related incorrect information (E. L. Bjork, Bjork, & Anderson, 1998; R. A. Bjork, 1989). The active suppression of information from memory is classically explored using directed forgetting paradigms.

Directed forgetting (DF) refers to a deliberate attempt to limit the future expression of specific memory contents (Johnson, 1994). DF has traditionally been investigated through the use of two distinct paradigms: the item and the list methods. In the item method, participants learn a list of items (study phase), with the instruction to remember every item followed by a “remember” cue (to-be-remembered items, TBR) and to forget items followed by a “forget” cue (to-be-forgotten items, TBF). Typically, in the subsequent test phase, TBR items are better recalled and recognized by comparison to TBF items: the so-called directed forgetting effect (Basden & Basden, 1998; MacLeod, 1975, 1998). Two main hypotheses have been proposed to explain the DF effect in the item method. First, the selective rehearsal account (Basden, Basden, & Gargano, 1993; R. A. Bjork & Woodward, 1973a) assumes that TBR items are more deeply encoded than TBF items (through rehearsal), making them more easily accessible for later remembering. According to this hypothesis, when an item is followed by a “remember” cue, participants typically engage in rehearsal and more elaborated encoding than when items are followed by a “forget” cue. Second, the attentional inhibition hypothesis (Zacks & Hasher, 1994; Zacks, Radvansky, & Hasher, 1996) states that the presentation of the forget instruction suppress the processing and rehearsal of TBF items (attentional inhibition), thus preventing working memory overload from irrelevant information and allowing more elaborated processing of TBR information (e.g., better selective rehearsal). According to this hypothesis, TBF items and/or the rehearsal of these items are assumed to be inhibited just after they are encoded (when the “forget” cue is displayed).

In agreement with the postulated differential encoding between TBR and TBF words (i.e., selective rehearsal, attentional inhibition) during the item method, it has recently been demonstrated with functional neuroimaging (Bastin et al., 2012) that a complex interplay of cognitive processes operates on TBR and TBF items in order to generate the directed forgetting effect. Indeed, successful encoding and retrieval of TBR items engage a set of regions well known to support deep and associative encoding and retrieval processes in episodic memory (the entorhinal cortex, the hippocampus, the anterior medial prefrontal cortex, the left inferior parietal cortex, the posterior cingulate cortex and the precuneus). In contrast, encoding of items to forget is associated with higher activity in regions known to intervene in attentional/executive control (the right middle frontal and posterior parietal cortex), and the correct recognition of these items at retrieval yields activation in regions associated with familiarity-based memory processes (the dorsomedial thalamus) and top-down attentional processes (posterior intraparietal sulcus and anterior cingulate cortex). In the same vein, Rizio and Dennis (2013) showed that encoding-related processes in the left inferior PFC and medial-temporal lobe contribute to subsequent memory success, whereas inhibitory processes in the right superior frontal gyrus and right inferior parietal lobe contribute to subsequent forgetting success.

On this basis, it was proposed (Bastin, et al., 2012) that when a word is followed by a “remember” cue, participants could engage articulatory rehearsal, facilitating the establishment of elaborative encoding. Further, TBR items undergo effortful associative encoding into long term memory that leads, at retrieval, to the reactivation of the rich memory trace created at encoding, a trace which includes the information itself associated with contextual details (a “recollection” process). In contrast, when a word is labelled “to forget”, cognitive processes related to the selection of information to enter short-term memory come into play because the replacement of information encoding by suppression/selection processes becomes mandatory. Hence, TBF items probably undergo only minimal superficial encoding, so that old TBF items are difficult to discriminate and successful retrieval of TBF happens mainly when the participant merely feels the item was familiar, as suggested by the activation of brain regions involved in familiarity processes and top-down attentional processes during memory retrieval. In agreement with that proposal, studies that used the Remember-Know-Guess (RKG) procedure [(Tulving, 1985), (see Gardiner, Ramponi, & Richardson-Klavehn, 2002, for a meta-analysis)] showed that participants’ subjective experiences during recognition decisions differ for TBR and TBF items. Indeed, more Remember judgments (which reflect conscious recollection of the encoding episode) have been associated with TBR than TBF information, contrary to Know judgments (reflecting a feeling of familiarity about the information), which did not differ between both types of item (Basden & Basden, 1996b; Gardiner, Gawlik, & Richardson-Klavehn, 1994).

The literature on directed forgetting in normal aging that used the item method evidenced an age-related decline (e.g., Collette, Germain, Hogge, & Van der Linden, 2009a; Dulaney, Marks, & Link, 2004a; Hogge, Adam, & Collette, 2008; Sego, Golding, & Gottlob, 2006; Zacks, Radvansky, et al., 1996) in the majority of studies (for a review, see Titz & Verhaeghen, 2010). For example, Zacks, Hasher and Radvansky (1996) et al. found a smaller DF effect for older than younger participants in a recall task. They interpreted this finding in reference to their more general hypothesis of inhibitory decline with the advance in age (Hasher & Zacks, 1988). More specifically, this DF impairment would originate from difficulties in inhibiting the processing of irrelevant information (i.e., TBF words) once the forget instruction was presented. Concerning recognition performance, the effects of aging are less clear; some authors evidenced a reduction in the size of the DF effect (Dulaney, Marks, & Link, 2004b; Zacks, Hasher, et al., 1996), whereas others found a DF effect of similar amplitude in both groups, despite a globally poorer recognition performance in older adults (Sego, Goldbing, & Gottlob, 2006). Up to now, no study examined RKG judgments associated with TBR or TBF information with the advance in age.

Interestingly, Gamboz and Russo (2002) suggested that the smaller DF effect observed in older participants with the item method “may reflect larger age-related differences in recall of words processed extensively (the TBR words) compared to recall of words processed only superficially (the TBF words), occurring as a consequence of the well documented age-related episodic memory deficit” (Gamboz & Russo, 2002, p. 367). Hence, for the authors, age-related inhibitory deficit is not the best candidate to explain the reduction of the DF effect in normal aging. Along those lines, using a processing level manipulation with the item method, they showed larger DF effects for younger than older participants in the shallow processing (to count the number of letters in the word) and control (no processing instructions) conditions, but not in the deep processing condition (to judge the pleasantness of each word), in which the older group experienced an equivalent DF effect to that of younger adults, although they recalled overall fewer TBR and TBF words. The authors argued that when both TBR and TBF words were processed extensively, both groups manifested equivalent DF effects due to the operation of similar inhibitory processes between groups. However, an important limitation of that study was that performance of older adults was unchanged across processing level conditions, so that there was no evidence of improved quality of memory traces in older adults. Moreover, the equivalent DF effect between groups in the deep processing condition came from the increased recall rate of TBF words for younger adults. Finally, there exist divergent findings, as another study using a similar processing manipulation (Dulaney, et al., 2004b) failed to evidence any effect of processing level manipulation on DF performance of young and older participants.

### Memory-trace quality as an alternative account of age-related smaller DF effect

As an alternative to the classical interpretation of smaller DF effect in aging in term of inhibitory deficit, the present study aimed at investigating the role of age-related differences in quality of memory traces on the magnitude of DF effects. More specifically, we hypothesize that, due to their well-known episodic memory deficits, and more particularly to a decline in the ability to self-initiate spontaneously deep and elaborate encoding strategies (Bouazzaoui et al., 2010; Craik & Rose, 2012; Saczynski, Rebok, Whitfield, & Plude, 2007), older participants would present weaker memory traces for the information they just encoded compared to younger adults. As a consequence, inhibitory processes, supposed to apply on some of those memory traces (i.e., TBF information), will require less effort. Therefore, in addition to a standard encoding condition, we submitted older participants to an encoding condition that improves the quality of memory traces by increasing presentation time of each item and providing strategies known to lead to a better encoding of the information. In that way, memory performance for TBR information should be equated between young and older participants, so that we can investigate inhibitory mechanisms in older participants when they apply to memory traces of similar strength as those of younger participants. In addition to the classical recall task, the DF effect was also investigated with a recognition task. This procedure allowed us a more qualitative assessment of the DF effect in aging by comparing inhibition abilities in conditions varying the requirement of self-initiated retrieval processes. Indeed, it has been argued that older people may be particularly disadvantaged on tasks requiring self-initiated processes such as recall tasks (Craik & Byrd, 1982). In addition, the recognition procedure allows the production, for each “yes” response provided, of a RKG judgment (Tulving, 1985). This will allow us to investigate participants’ subjective experiences accompanying their recognition decisions.

Our main prediction was that improving the quality of memory traces will modify the age-related effect on directed forgetting when compared to standard (short) encoding conditions. First, in the standard/short encoding condition and for the recall task, we expected a significant DF in the two groups, although smaller for older participants, a result reported several times previously [e.g., (Sego, Goldbing, et al., 2006; Zacks, Hasher, et al., 1996)]. The rationale here was that, due to their weaker memory traces, the remaining inhibitory abilities of older participants would be enough to prevent the processing of TBF words associated with poorer memory traces, creating the observed significant DF effect. But it would appear reduced because TBR items would also have weaker memory traces in older participants. A similar prediction was made for the recognition task, although results are less clear in the literature, with some authors showing a smaller DF effect in aging (Dulaney, et al., 2004b; Zacks, Hasher, et al., 1996), while others don’t (Sego, Goldbing, et al., 2006). With regard to the subjective experience of recognition, we expected more R responses associated to TBR than TBF words for both groups (see, for data on young subjects, Basden & Basden, 1996b; Gardiner, et al., 1994), despite a globally higher rate of R responses for younger adults, given the well-known age effect on recollection (e.g., Anderson et al., 2008; Bastin & Van der Linden, 2003; Prull, Dawes, Martin, Rosenberg, & Light, 2006). Finally, a larger DF effect for information associated to R responses is expected in younger (Basden & Basden, 1996b; Gardiner, et al., 1994) but not in older participants.

Second, we predicted that an increase in the quality of the memory traces of older participants (strong/long encoding condition) would require more inhibition to suppress TBF words processing. Therefore, a critical comparison will be the comparison between the standard/short encoding condition in young subjects and the strong/long encoding condition in older participants, which should match the quality of memory traces between the two groups. So, when their episodic memory performance equates that of younger participants, older adults should present no more or much reduced DF effect due to their inhibitory difficulties. Indeed, their limited inhibition abilities should be inefficient in the face of stronger TBF memory traces. Similarly, we also expected a disappearance of the DF effect for older participants in the recognition task. Finally, concerning RKG judgments, we should observe a suppression of the effect of aging on R responses (for both TBR and TBF), due to the equalization of the memory traces. Moreover, because of their decreased inhibitory abilities, older adults should report a similar proportion of R responses for both TBR and TBF words, leading again to a disappearance of the directed forgetting effect.

## Methods

### Participants

Forty-eight young and 48 older adults took part in this experiment, and were naive about the purpose of the experiment. The participants were arbitrarily attributed to a condition (standard versus strong encoding) so that each condition was administered to 24 young and 24 older participants. The demographic and cognitive characteristics of both groups in each condition are reported in Table 1. Participants did not differ between the conditions, except for vocabulary performance (French adaptation of the Mill Hill test (Deltour, 1993)), F(1, 92) = 5.14, p < .05, and for verbal memory performance, F(1, 92) = 6.22, p < .05, which were better in participants in the strong encoding condition. However, condition difference did not interact with age (ps > .68) and so should not influence future analyses.

**Table 1:** Group characteristics as a function of condition.

	Young		Older	

	Standard	Strong	Standard	Strong

Women / men	10 / 14	14 / 10	10 / 14	14 / 10

Age	22.0 (2.5)	22.1 (2.6)	68.9 (2.8)	68.2 (3.1)

Education	14.4 (1.8)	14.5 (1.7)	14.0 (1.7)	13.9 (2.0)

Mattis DRS	-	-	141.87 (1.68)	142.83 (1.31)

Mill Hill	22.75 (4.37)	24.67 (3.64)	27.29 (3.87)	28.83 (2.91)

Stroop				

Interference time	87.17 (13.44)	89.79 (17.29)	124.21 (32.69)	129.12 (33.07)

Errors	2.37 (1.76)	3.67 (3.38)	2.12 (3.01)	1.75 (1.75)

Interference index	.21 (.05)	.24 (.05)	.30 (.06)	.29 (.08)

Hayling				

Inhibition time	61.33 (23.87)	57.25 (27.81)	69.58 (12.58)	86.62 (51.76)

Inhibition errors	3.21 (2.28)	3.33 (1.93)	5.75 (2.89)	5.41 (4.11)

Digit span				

Forward	6.08 (0.92)	6.50 (1.10)	5.96 (1.08)	5.75 (1.03)

Backward	5.00 (1.10)	5.17 (1.34)	4.00 (1.32)	4.33 (1.24)

Verbal Memory	39.50 (3.41)	41.45 (3.08)	30.92 (6.38)	33.62 (4.72)

*Note*: Standard deviations in parentheses.

This study was performed in accordance with the ethical standards described in the Declaration of Helsinki (1964), and approved by the Ethics Committee of the Faculty of Psychology at the University of Liège. All participants were native French speakers, reported being in good health and having good (or corrected-to-normal) hearing and vision. They reported no history of medical, neurological or psychiatric disorders, and were not using any medications that could influence their performance during the tasks. The cognitive status of the older group was evaluated with the Mattis Dementia Rating Scale (Mattis, 1976). All had a total score equal to or greater than 130 (M = 142.35; SD = 1.56; range 138–144), which constitutes the cut-off score to distinguish between normal aging and dementia (Monsch et al., 1995).

### Materials

The materials included a list of 64 six-letters words (concrete nouns or verbs) selected from the Brulex French database (Content, Mousty, & Radeau, 1990). Within those 64 words, 16 served as TBR, 16 as TBF, and 32 as distracters for the recognition task. TBR and TBF items were presented pseudo-randomly, with the use of four versions of the task, which were counterbalanced across participants (words that were TBR in one version were TBF in another and words that were targets during the study phase in one version served as distracters during recognition phase in another version). For each version, presentation order for the words was constant for all participants. Importantly, care was taken to ensure that each type of item could not be presented more than three times consecutively.

### Design

A 2 (Age group: young vs. older) x 2 (Item type: TBR vs. TBF) x 2 (Encoding: standard vs. strong) design was used in this experiment. Age group and encoding condition were between-participant factors, and item type was a within-participant factor.

### Procedure

Participants were first instructed that they would be presented with a list of words, each word followed by an instruction either “to remember” or “to forget”, and that the memory test would only concern TBR words. Importantly, they were instructed to process and encode every item in a similar fashion as soon as they appeared on the screen and until the presentation of the TBR/TBF cue. Moreover, in the strong encoding condition only, the use of three possible mnemonic strategies was suggested to the participants before performing the DF task: rote repetition, sentence generation, and mental imagery. For the repetition strategy, participants were explained that they could repeat as many times as they wanted the presented word. For the sentence generation, they were instructed to create a sentence using the word to remember. For the mental imagery strategy, they were instructed to imagine a picture containing the word to remember. Finally, participants were also told that they could use any other mnemonic strategy that they judge useful for memorization. This procedure is similar to the one used by Froger, Bouazzaoui, Insigrini and Taconnat (2012) which showed that the performance of older participants is improved when instructions about mnemonic strategies are provided and encoding time increased.

During the study phase, 32 words were individually presented in the center of the screen during 5 s (or 9 s in the strong encoding condition), each one immediately followed by the instruction to either forget (“to forget”) or remember (“to remember”) that word, displayed during 3 s. After the memory cue, the screen remained black for 1 s. A fixation cross was displayed before each word for a duration of 1500 ms. Once the entire list was presented, participants performed a distracter arithmetic task during 30 s in which they had to count backward in steps of 3 in order to suppress any recency effect. Immediately after that task, participants were asked to recall and then to recognize as many words as possible from the study phase, regardless of whether they were associated with a remember or a forget cue. For the recall task, participants gave their responses orally in any order (duration of at least 120 s). In the recognition task, the 32 target words (16 TBR and 16 TBF) were presented individually on the screen intermixed with 32 distracter words in a random order. Each word was presented until the participant made his recognition judgment orally (yes/no), and the experimenter pressed the corresponding key on the keyboard. In addition, for each recognized word (or false alarm recognition), participants were asked to provide a Remember/Know/Guess (RKG) judgment. They must give a “Remember” judgment (R) each time they were sure of having encountered the item in the study phase and could recollect any aspect of the encoding context (conscious recollection); a “Know” judgment (K) each time they were sure that the item was previously encountered without being able to recollect any learning context detail (familiarity); and a “Guess” judgment (G) each time they were unsure that the word had appeared. To be sure that the participants correctly understood the difference between the three kinds of judgments, they were given examples of RKG judgments by the experimenter. In addition, they were systematically asked to explain their judgments for the first words of the recognition task.

Two inhibitory tasks were also administered to participants: the Stroop (Stroop, 1935) and Hayling (Burgess & Shallice, 1996) tasks. In the Stroop task, subjects are confronted with words written in different colors and are asked to name the colors as quickly as possible while ignoring the words themselves. A response time (RT) interference score was calculated by comparing performance in that interference condition to a naming condition in which subjects have to name colored squares using the formula: (interference – naming) / (interference + naming). In the Hayling task, sentences in which the final word is omitted, but has a particularly high probability of one specific response, are presented. In section A (initiation), subjects have to complete the sentence with the missing word. In section B (inhibition), subjects have to complete the sentence not with the expected word but with a word unrelated to the sentence. Performance in the inhibition condition was assessed by response time and semantic relatedness of the response to the missing word.

## Results

### Free recall

**Figure 1:**
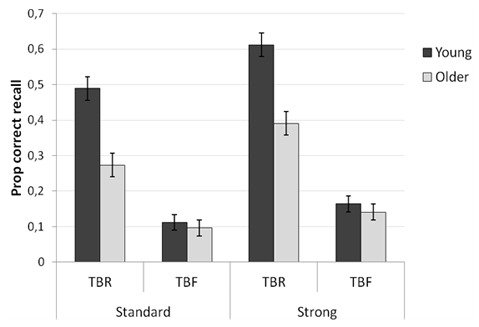
Mean proportions of correctly recalled TBR and TBF items as a function of age group and encoding condition.

The mean proportions of TBR and TBF words that were correctly recalled are presented in Figure 1. The 2 (Age group: young vs. older) x 2 (Encoding: standard vs. strong) x 2 (Item type: TBR vs. TBF) repeated measure ANOVA evidenced a main effect of age group [*F*(1, 92) = 34.46; *p* < .001, *η*^2^*_p_* = .27], of encoding [*F*(1, 92) = 17.12; *p* < .001, *η*^2^*_p_* = .16], and of item type [*F*(1, 92) = 253.21; *p* < .001, *η*^2^*_p_* = .73], indicating that young participants recalled more words than older participants, that participants recalled globally more words in the strong encoding condition, and that participants recalled globally more TBR than TBF words. In addition, the age group x item type interaction was significant [*F*(1, 92) = 25.62; *p* < .001, *η*^2^*_p_* = .22]. HSD Tukey tests indicated that, whereas young participants recalled more TBR words than older participants (p < .001), there was no age-related difference in the proportions of recalled TBF words. This interaction thus points to a reduction of the amplitude of directed forgetting effect in aging related to a reduced recall performance of TBR items. No other interaction reached significance.

In order to check that the strong encoding condition was efficient in matching older adults’ memory performance to that of young adults in the standard encoding condition, we directly compared performance of younger adults in the standard encoding condition to that of older participants in the strong encoding condition by means of a 2 (Age group: young vs. older) x 2 (Item type: TBR vs. TBF) repeated measure ANOVA. This analysis showed that there was only a main effect of item type [*F*(1, 46) = 96.14; *p* < .001, *η*^2^*_p_* = .67], with both groups recalling more TBR than TBF words. There was no main effect of group [*F*(1, 46) = 1.89; *p* > .17, *η*^2^*_p_* = .04], and no interaction [*F*(1, 46) = 3.97; *p* > .05, *η*^2^*_p_* = .08].

Finally, we also checked whether the manipulation of encoding effectively improved the memory performance of older adults by means of a 2 (Encoding: standard vs. strong) x 2 (Item type: TBR vs. TBF) repeated measure ANOVA. The analysis showed an effect of encoding [*F*(1, 46) = 10.07; *p* < .005, *η*^2^*_p_* = .17], with a better performance in the strong encoding condition, a main effect of item type [*F*(1, 46) = 70.17; *p* < .001, *η*^2^*_p_* = .670], with a better performance for TBR information, but no interaction [*F*(1, 46) = 2.05; *p* > .05, *η*^2^*_p_* = .04].

### Recognition memory

**Figure 2:**
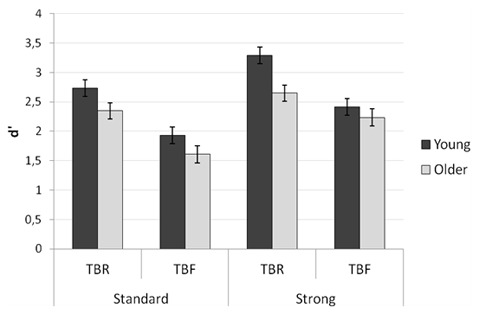
Recognition accuracy (discrimination d’ score) of TBR and TBF items as a function of age group and encoding condition.

Proportions of “old” responses to TBR, TBF and new items for young and older adults are presented in Table 2. The ability to correctly discriminate studied items (either TBR or TBF) from new items was indexed by d’ scores (Macmillan & Creelman, 1991). The discrimination d’ scores for each group in each encoding condition are presented in Figure 2. These scores were submitted to a 2 (Age group: young vs. older) x 2 (Encoding: standard vs. strong) x 2 (Item type: TBR vs. TBF) repeated measure ANOVA. The results yielded a main effect of age group [*F*(1, 92) = 8.66; *p* < .01, *η*^2^*_p_* = .08], of encoding [*F*(1, 92) = 14.38; *p* < .001, *η*^2^*_p_* = .13], and of item type [*F*(1, 92) = 160.86; *p* < .0001, *η*^2^*_p_* = .63], indicating that young participants recognized more words than older participants, that both groups recognized more words in the strong encoding condition than in the standard encoding condition, and that participants recognized more TBR than TBF words. There was also a significant age group by item type interaction [*F*(1, 92) = 5.45; *p* < .05, *η*^2^*_p_* = .0.05]. Post-hoc Tukey tests revealed that young participants had a greater capacity to discriminate TBR items from new items than older adults (p < .01), whereas there was no group difference for TBF discrimination d’ score (p > .28). Hence, as for recall performance, these results suggest that young participants had a stronger directed forgetting effect than older adults, driven by better memory performance for TBR items.

**Table 2:** Mean proportions of old responses to TBR, TBF and new items as a function of age group and encoding condition.

	Standard		Strong	

	Young	Older	Young	Older

TBR	.87 (.11)	.67 (.23)	.93 (.06)	.79 (.15)

TBF	.65 (.18)	.43 (.22)	.72 (.14)	.66 (.21)

New	.10 (.11)	.04 (.05)	.04 (.05)	.05 (.06)

*Note*: Standard deviations in parentheses.

Additionally, we compared discrimination d’ scores obtained by young participants in the standard encoding condition to that obtained by older participants in the strong encoding condition by means of a 2 (Age group: young vs. older) x 2 (Item type: TBF vs. TBF) repeated measure ANOVA. The analysis indicated that there was no main effect of age group [*F*(1, 46) = 0.30; *p* > .58, *η*^2^*_p_* = .006]. The main effect of item type was significant [*F*(1, 46) = 50.83; *p* < .001, *η*^2^*_p_* = .52], indicating greater recognition accuracy for TBR than TBF items. The age group by item type interaction was significant [*F*(1, 46) = 5.22; *p* < .05, *η*^2^*_p_* = .10], but post-hoc Tukey tests did not reveal any group difference for the capacity to recognize TBR items (p > .97) or TBF items (p > .50). And both groups showed a directed forgetting effect (ps < .01).

Finally, we also checked that the manipulation of encoding effectively improved the discrimination scores of older adults by means of a 2 (Encoding: standard vs. strong) x 2 (Item type: TBR vs. TBF) repeated measure ANOVA. The analysis showed an effect of encoding [*F*(1, 46) = 5.11; *p* < .05, *η*^2^*_p_* = .09], with a better performance in the strong encoding condition, a main effect of item type [*F*(1, 46) = 45.04; *p* < .001, *η*^2^*_p_* = .49], with a better performance for TBR information, but no interaction [*F*(1, 46) = 3.07; *p* > .05, *η*^2^*_p_* = .07].

### Remember, Know, and Guess judgments

Remember, Know, and Guess (RKG) judgments accompanying the correct recognition of TBR and TBF items or given to new items (false alarms) are presented in Table 3.

**Table 3:** Mean proportions of RKG judgments of both groups as a function of item type and encoding condition.

		Standard		Strong	

		Younger	Older	Younger	Older

TBR	*Remember*	.54 (.26)	.45 (.26)	.76 (.21)	.55 (.28)

	*Know*	.25 (.17)	.14 (.13)	.10 (.10)	.19 (.21)

	*Guess*	.07 (.08)	.08 (.10)	.07 (.12)	.04 (.06)

TBF	*Remember*	.25 (.19)	.21 (.20)	.45 (.22)	.39 (.27)

	*Know*	.23 (.15)	.12 (.12)	.17 (.15)	.19 (.16)

	*Guess*	.16 (.10)	.09 (.07)	.10 (.09)	.07 (.07)

New	*Remember*	.01 (.03)	.005 (.01)	0	.006 (.01)

	*Know*	.03(.04)	.01 (.02)	.02 (.03)	.01 (.03)

	*Guess*	.05 (.06)	.02 (.04)	.03 (.03)	.03 (.05)

*Note*: Standard deviations in parentheses.

For the analysis of correct responses, a 2 (Age group: young vs. older) x 2 (Encoding: standard vs. strong) x 2 (Item type: TBR vs. TBF) repeated measure ANOVA was performed on the proportions of each type of judgments. For R judgments, the ANOVA revealed a main effect of age group [*F*(1, 92) = 5.21; *p* < .05, *η*^2^*_p_* = .05], showing more Remember judgments in the young group compared to the older group; a main effect of encoding condition [*F*(1, 92) = 15.87; *p* < .001, *η*^2^*_p_* = .14], showing that there were more R responses after encoding the words for 9 s than after a 5s-encoding; and a main effect of item type [*F*(1, 92) = 128.36; *p* < .001, *η*^2^*_p_* = .58], indicating that participants gave more R judgments to TBR than TBF items. The age group by item type interaction was also significant [*F*(1, 92) = 5.35; *p* < .05, *η*^2^*_p_* = .05]. Post-hoc Tukey tests revealed that young participants gave more R judgments to TBR items than older participants (p < .05), but not to TBF items (p > .75). For K judgments, the ANOVA showed that there was an age group by encoding condition interaction [*F*(1, 92) = 9.85; *p* < .01, *η*^2^*_p_* = .09]. This interaction was due to the fact that, in the standard encoding condition, young participants gave globally more Know responses than older participants (p < .05), but there was no group difference after 9s of encoding (p > .43). No other effect was significant. Finally, for Guess responses, the analysis showed a main effect of encoding condition [*F*(1, 92) = 4.10; *p* < .05, *η*^2^*_p_* = .04], with more Guess responses after a 5s-encoding than after 9s of encoding, a main effect of item type [*F*(1, 92) = 14.29; *p* < .001, *η*^2^*_p_* = .13], with more Guess judgments to TBF items than to TBR items, and no other significant effect.

Given the very low proportions of incorrect Remember responses (R responses to new words), which were around 1%, they were not analyzed. Finally, 2 (Age group: young vs. older) x 2 (Encoding: standard vs. strong) ANOVAs on incorrect Know and Guess responses showed no significant effect (ps > .09).

The final set of analyses examined RKG judgments to TBR and TBF items when global recognition accuracy was matched between groups (i.e., when the young group studied the words for 5 s and the older group for 9 s). The 2 (Age group: young vs. older) x 2 (Item type: TBR vs. TBF) repeated measure ANOVA on the proportion of R judgments showed only a main effect of item type [*F*(1, 46) = 38.68; *p* < .001, *η*^2^*_p_* = .46], indicating a greater amount of R judgments for TBR than TBF items in both groups. For K judgments, there was no significant effect (ps > .24). Finally, for G judgments, a main effect of group [*F*(1, 46) = 11.85; *p* < .01, *η*^2^*_p_* = .20] and of item type [*F*(1, 46) = 15.57; *p* < .001, *η*^2^*_p_* = .25] emerged. Hence, young adults reported globally more G judgments than older participants; and there were more Guess responses to TBF words than to TBR words. Given that differences in the proportion of Guess may reflect changes in response bias, we performed an Age group by Item type ANOVA on response criterion C. It revealed no main effect of age group [F(1,46) = 2.64, p = .11, *η*^2^*_p_* =.05], a significant main effect of item type [F(1,46) = 50.83, p <0.001, *η*^2^*_p_* = .52] and a significant interaction [F(1,46) = 5.23, p <0.05, *η*^2^*_p_* =. 10] due to a slightly larger effect of item type in young than in older participants. Altogether, these results indicated that, when memory performance is matched for TBR items between groups, both young and older adults showed a comparable directed forgetting effect, which appeared only for Remember responses.

Finally, we also checked whether the manipulation of encoding effectively modified RKG judgments of older adults by means of a 2 (Encoding: standard vs. strong) x 2 (Item type: TBR vs. TBF) repeated measure ANOVA. For Remember responses, the analysis showed an effect of encoding [*F*(1, 46) = 4.49; *p* < .05, *η*^2^*_p_* = .09], with more R responses in the strong encoding condition, a main effect of item type [*F*(1, 46) = 45.17; *p* < .001, *η*^2^*_p_* = .49], with a better performance for TBR information, but no interaction [*F*(1, 46) = 1.71; *p* > .05, *η*^2^*_p_* = .03]. No significant effects were observed for Know and Guess responses.

### Inhibition measures and directed forgetting

First, the performance of the young and older groups was compared on measures of inhibition: Stroop test (time and errors in the interference condition; interference index calculated as the difference between time to complete the interference condition minus time to complete the color naming condition, divided by their sum) and Hayling task (time and errors in the inhibition condition). Each group’s mean performance as a function of the experimental condition (standard versus strong encoding) is presented in Table 1. Age group by Encoding condition ANOVAs performed on these scores did not reveal any difference between conditions, nor any age by condition interaction. However, age-related differences were observed in the Stroop test (time to complete the interference condition, F(1, 92) = 52.99, p < .001; interference errors, F(1, 92) = 4.21, p < .05; interference index, F(1, 92) = 27.65, p < .001), and in the Hayling task (time to complete the inhibition condition, F(1, 92) = 7.43, p < .01; inhibition errors, F(1, 92) = 14.99, p < .001). Moreover, older participants also performed poorer than young participants on working memory measures: forward digit span, F(1, 92) = 4.59, p < .05, and backward digit span, F(1, 92) = 20.17, p < .001, and on verbal memory, F(1, 92) = 27.65, p < .001. For the sake of completeness, correlations were computed between inhibition measures in each group. The only significant correlation was between Hayling time and Stroop interference index in the older group (r = .37, p < .05).

In order to assess whether the amplitude of the directed forgetting effect in recall and recognition tasks correlated with participants’ inhibition capacities, Pearson correlations were computed between the measure [score for TBR items - score for TBF items] in the recall and recognition parts and inhibition measures for each group and each encoding condition. In young participants, the results did not reveal any significant correlation (ps > .24). In older adults, the only significant correlations emerged for participants in the strong encoding condition between the amplitude of the directed forgetting effect in recall and time to complete the inhibition condition in the Hayling task (r = -.41, p < .05). So, older participants who needed more time to complete the sentences with an unrelated word had a smaller directed forgetting effect.

In order to assess if DF effects differ according to the inhibition abilities (as suggested by the correlations between that measure and performance on the Hayling tasks in older), our groups of participants were subdivided into subgroups according to their performance on the Hayling task (RTs above and below the median). With regard to the standard encoding condition, 2 (Group: high vs. low inhibitory score) x 2 (Item type: TBR vs. TBF) repeated measure ANOVAs were performed. For recall performance, the analysis showed a main effect of item type [*F*(1, 22) = 42.80; *p* < .001, *η*^2^*_p_* = .66], with a better performance for TBR information, but no effect of group [*F*(1, 22) = 0.24; *p* > .05, *η*^2^*_p_* = .01], nor interaction [*F*(1, 22) = 0.77; *p* > .05, *η*^2^*_p_* = .03]. A similar pattern of response was observed for the d’ discrimination score, with only a main effect of the item type [*F*(1, 22) = 51.06; *p* < .001, *η*^2^*_p_* = .70; main group effect: *F*(1, 22) = 0.28; *p* > .05, *η*^2^*_p_* = .01; interaction: *F*(1, 22) = 0.005; *p* > .05, *η*^2^*_p_* = .0002]. The same ANOVA performed for the strong encoding condition showed, for recall performance, a main effect of item type [*F*(1, 22) = 31.38; *p* < .001, *η*^2^*_p_* = .58], with a better performance for TBR information, but no effect of group [*F*(1, 22) = 0.16; *p* > .05, *η*^2^*_p_* = .007], nor interaction [*F*(1, 22) = 0.22; *p* > .05, *η*^2^*_p_* = .01]. A similar pattern of response was observed for the d’ discrimination score, with only a main effect of the item type [*F*(1, 22) = 7.75; *p* < .05, *η*^2^*_p_* = .26; main group effect: *F*(1, 22) = 0.11; *p* > .05, *η*^2^*_p_* = .004; interaction: *F*(1, 22) = 0.13; *p* > .05, *η*^2^*_p_* = .005].

## Discussion

In the current study, we compared young and older adults’ performance on directed forgetting (DF) tasks using the item method. Two encoding conditions were administered (standard/short encoding vs. strong/long encoding) to determine the efficiency of the DF inhibitory process in normal aging. When memory performance was not equated between participants, the results indicated smaller amplitude of the DF effect in aging related to reduced recall/recognition performance of TBR items. Moreover, the RKG procedure showed a larger contribution of recollection process to the retrieval of TBR information in young than older adults. However, when the memory trace was equated between groups (comparison of the standard encoding condition in young to the strong encoding condition in older participants), DF effects of similar amplitude were observed, for both the recall and recognition tasks, and recollection processes contributed in the same way to the DF effect in young and older participants.

Our first prediction stated that, when young and older participants are submitted to similar encoding condition, older adults would present a smaller DF effect than younger adults (i.e., smaller difference between TBR and TBF words recall). This is a finding classically associated with aging in the directed forgetting literature (Collette, Germain, et al., 2009a; Dulaney, et al., 2004a; Hogge, et al., 2008; Sego, Golding, et al., 2006; Zacks, Radvansky, et al., 1996). In this line, the results confirmed our hypothesis. Indeed, despite older adults manifesting a significant forgetting, this effect was of smaller amplitude compared to younger, as attested by their lower recall of TBR words and equivalent recall of TBF words. Interestingly, a smaller DF effect is also observed when considering recognition data; a pattern that has already been reported, but not consistently, in the literature [(Dulaney, et al., 2004b; Zacks, Hasher, et al., 1996); see however (Sego, Goldbing, et al., 2006)]. The presence of smaller DF effects in both recall and recognition tasks in aging is consistent with the view that the mainstay of the age-related differences in item method DF is situated at encoding. Indeed, the item method DF has been associated with differential encoding, selective rehearsal, partitioning of items and attention inhibition [see (Titz & Verhaeghen, 2010)]. These mechanisms are all assumed to operate at encoding and are, to the exception of rehearsal, impaired by normal aging (Craick & Salthouse, 2008).

In addition, the analyses of RKG judgments brought some interesting results. First, we observed that, in the two groups, more Remember judgments were associated with TBR than TBF information, contrary to Know judgments, which did not differ between both types of items (for similar results, see (Basden & Basden, 1996a; Gardiner, et al., 1994)). According to the selective encoding hypothesis (R. A. Bjork, 1970; R. A. Bjork & Woodward, 1973b), each item is maintained in active memory until the cue is presented. If the cue is to remember, then the item is processed further, whereas if the cue is to forget, the item is dropped from active memory and is not processed further. Thus, TBR information should undergo more elaborated encoding than TBF information, which receives shallow encoding. The current finding of selective increase of recollection for TBR information is consistent with previous evidence of predominant enhancement of recollection following deep encoding compared to shallow encoding (Yonelinas, 2002, for a review).

Second, the inclusion of RKG judgments in this study qualified for the first time the nature of the memory traces of young and older adults during directed forgetting. The results indicated that the reduction of the directed forgetting effect in normal aging is driven by differences in recollection, but not in familiarity. Larger age-related effects on recollection than familiarity are typically observed in memory tasks (e.g., Anderson, et al., 2008; Bastin & Van der Linden, 2003; Prull, et al., 2006). Interestingly, here, the recollection decline is only evidenced for TBR information. In the context of the selective encoding hypothesis, this would indicate that older participants failed to engage into effortful elaboration processes susceptible to induce recollection for TBR words. This difficulty may come from their reduced capacity to self-initiate deep encoding strategies (Bouazzaoui, et al., 2010; Craik & Rose, 2012; Froger, et al., 2012; Saczynski, et al., 2007). An alternative, but not mutually exclusive, interpretation may be that age-related differences in recollection arose because the short time of presentation in the standard condition prevented older participants to encode source information related to the words in a sufficiently distinctive way. Indeed, because of reduced speed of processing in aging (Clarys, Isingrini & Gana, 2002), older participants may have encoded the TBR versus TBF status of each word, but could not elaborate a sufficient amount of associated information to make TBR richly remembered in the subsequent memory test. In contrast, older adults seemed to process TBF words in a way that was comparable to that of young participants. Altogether, this supports the idea that previous findings of smaller DF effects in aging may result from impoverished processing of TBR information, so that one cannot conclude about the influence of inhibitory processes in these studies.

The central hypothesis of the present study was that older adults would evidence decreased DF abilities when matched to younger adults with regard to memory performance for TBR information, due to their less efficient inhibitory functioning. Although the encoding manipulation lead to an effective increase of memory performance in the older group, the results failed to support this prediction. Indeed, we did not evidence a reduced DF effect for older participants when equated to younger for TBR items performance (comparison of older group in the strong vs. younger group in the standard encoding condition) for both recall and recognition. Moreover, the DF effect was similarly driven by Remember responses in young and older participants. So, the results indicated that increasing the quality of memory traces in older participants by improving elaborate encoding and subsequent recollection of TBR information led to equivalent DF effects in both groups. Hence, contrary to other item-method studies, which argued for a decrease in inhibitory abilities of older adults (e.g. Dulaney et al., 2004; Zacks et al. 1996), our results argue in favor of a preservation of these inhibitory abilities, or, at least, suggest that DF in normal aging does not crucially depend on inhibitory abilities, but mainly depends on the selective processing of TBR information (e.g., through rehearsal and elaborated encoding). Accordingly, the comparison of older participants with high or low inhibitory abilities showed the presence of similar DF effects. These results agree with the proposition of Gamboz and Russo (2002) that age-related differences with the item method may mainly reflect age-related differences in the recall of words processed extensively at encoding (i.e., TBR words) rather than differences in inhibition.

In that context, two previous studies on normal aging reported a relative independence of memory and inhibition processes in item-method DF tasks. Salthouse et al. (2006) observed that controlling for age-related differences in TBR scores reduced semi-partial correlations between age and TBF scores essentially to zero. This implies that age-related effects in directed forgetting might be largely attributable to age differences in how TBR items are processed and recalled. More recently, Collette et al. (2009) separated their older participants in two groups according to their memory performance on TBR items and showed that the mean recall performance for TBF words was equivalent in the group that recalled a high percentage of TBR words and in the group that recalled a low percentage of TBR words.

However, a recent meta-analysis (Titz & Verhaeghen, 2010) showed the persistence of reliably smaller DF effect in older adults, even after controlling for age differences in baseline recall. Consequently, these authors argued that the age-related DF impairment cannot be reduced to a more general age-related problem in memory performance, but is also compatible with an inhibitory account of age effects in directed forgetting. In agreement with that proposal, we observed a significant correlation between the amplitude of the DF effect in recall and time completion in the Hayling task (Burgess & Shallice, 1996) for older adults in the strong encoding condition only, with the older participants who needed more time to complete the sentences with an unrelated word having a smaller DF effect. The Hayling task requires suppressing from working memory a word strongly activated by the sentence context, which is globally similar to the inhibition of the item when the Forget cue is presented after a long encoding period. Consequently, we cannot totally reject the hypothesis that a reduction of inhibitory mechanisms has a minimal impact on the DF effects of older participants in the present study. The use of hierarchical linear regression analyses should allow to better emphasize the respective contribution of memory and inhibition to the DF effect, and to examine if this contribution varies with advance in age. However, our sample size is not sufficient to perform such an analysis.

Finally, as normal aging was associated with impairment of controlled inhibitory processes and a preservation of automatic ones (Collette, Germain, et al., 2009b; Collette, Schmidt, Scherrer, Adam, & Salmon, 2009), we could also suggest that our DF procedure is not enough resource demanding to evidence a clear inhibitory dysfunction in the older participants. Indeed, Lee and Lee (2011) showed a deleterious effect of divided attention (backward counting during the presentation of TBR and TBF cues) on the recall of TBR, but not TBF, information in young adults. These results indicate that the suppression process in the item-method DF task require relatively few attentional resources, and may mainly consist in ignoring irrelevant information rather than to implement active suppression processes. Consequently, using procedures which decrease the attentional resources available or increase the difficulty to suppress irrelevant information should allow to better evidence the potential influence of inhibitory abilities on the DF effect in normal aging.

The current study adopted a cross-sectional design. As stressed by Nilsson (2012), longitudinal approaches allow a better characterization of cognitive aging, avoiding possible cohort effects. Therefore, future work should consider longitudinal changes in the interplay between inhibition and memory processes, also considering variables that contribute to individual variability in trajectories of cognitive aging. In particular, factors that contribute to building a cognitive reserve that attenuates the impact of age on cognition should be considered. This includes education and occupational attainment, social interactions and genetic characteristics.

## Conclusion

As a whole, the results of this experiment using the item method did not evidence, contrary to our main hypothesis, a decrease in directed forgetting abilities with the advance in age when both groups were equated for TBR memory performance. Indeed, when controlling for episodic memory differences between young and older participants, we did observe a DF effect of similar amplitude in both groups. These findings thus showed that older adults were as able as younger adults to efficiently suppress processing of words cued to forget. Hence, the smaller DF effect observed in the standard encoding condition, which is often reported in the literature and classically interpreted as an inhibitory failure (i.e., decreased ability to suppress TBF words) may rather reflect differences in episodic memory functioning.

## Author information

Dr. Fabienne Collette, Cyclotron Research Centre, 8 Allée du VI août (B30), B-4000 Liège, BELGIUM, Phone: +32 4 366 23 69, Email: f.collette@ulg.ac.be

Julien Grandjean, Cyclotron Research Centre, 8 Allée du VI août (B30), B-4000Liège, BELGIUM, Phone : +32 4 366 54 75, Email : julien.grandjean@ymail.com

Caroline Lorant, Cyclotron Research Centre, 8 Allée du VI août (B30), B-4000Liège, BELGIUM, Phone : +32 4 366 23 27, Email : caroline.lorant@doct.ulg.ac.be

Christine Bastin, Cyclotron Research Centre, 8 Allée du VI août (B30), B-4000Liège, BELGIUM, Phone : +32 4 366 23 27, Email : christine.bastin@ulg.ac.be
